# NOD2 function in Crohn's disease

**DOI:** 10.1186/1479-5876-9-S2-I10

**Published:** 2011-11-23

**Authors:** Alison Simmons

**Affiliations:** 1 Translational Gastroenterology Unit, Experimental Medicine Division – NDM, University of Oxford, John Radcliffe Hospital, Headington, Oxford; MRC Human Immunology Unit, Weatherall Institute of Molecular Medicine, John Radcliffe Hospital, Headington, Oxford, UK

## 

Abstract not submitted for publication

